# Draft genome sequence of the yeast *Schwanniomyces capriottii* strain UCD805, isolated from soil in Ireland

**DOI:** 10.1128/mra.01073-23

**Published:** 2024-02-05

**Authors:** Padraic G. Heneghan, Adam P. Ryan, Eoin Ó Cinnéide, Jordan Davies, Cathal Bracken, Victoria Ogundipe, Maria Doheny, Liam Lenihan, Anastasia Passalaris, Rosalind Walker, Kenneth H. Wolfe, Geraldine Butler, Kevin P. Byrne

**Affiliations:** 1 School of Medicine, Conway Institute, University College Dublin, Belfield, Ireland; 2 School of Biomolecular and Biomedical Science, Conway Institute, University College Dublin, Belfield, Ireland; University of California Riverside, USA

**Keywords:** yeasts, genome analysis

## Abstract

*Schwanniomyces capriottii* is a member of the Debaryomycetaceae family in the order Saccharomycetales. Here, we present the genome sequence of *S. capriottii* UCD805, which was isolated from soil in Dublin, Ireland. This genome is 12.2 Mb and was assembled into 14 scaffolds plus a mitochondrial genome scaffold.

## ANNOUNCEMENT

*Schwanniomyces capriottii* is a species in the Debaryomyces clade of the budding yeast family Debaryomycetaceae (1). It was previously known as *Debaryomyces castellii* but renamed in 2010 (2). It was first isolated from soil in Sweden (3). It has no known food or biotechnological applications, and as it does not grow at 37°C, it is unlikely to be a human pathogen.

*S. capriottii* UCD805 was isolated from soil collected on the University College Dublin campus in Dublin, Ireland (GPS coordinates 53.310884, –6.223159) as part of an undergraduate research module (4). The sample was collected from under a willow tree beside a forest walk.

Soil material was passaged twice at room temperature in 9 mL liquid yeast extract-peptone-dextrose (YPD) containing chloramphenicol (30 µg/mL) and ampicillin (100 µg/mL) and cultured on YPD agar plates. The species was identified by PCR and Sanger sequencing of the ribosomal DNA internal transcribed spacer (ITS) and D1/D2 regions (accession numbers OR578565 and OR578569). Sequence identity was 100% (591/591 bp) in the ITS and 99% (568/571 bp) in the D1/D2 region, to the type strain of *S. capriottii* (accession numbers KY105380 and KY109604).

DNA for genome sequencing was isolated from liquid YPD cultures grown at 20°C. For short-read sequencing, DNA was isolated by phenol/chloroform extraction. Illumina library construction (300-cycle v1.5 kit) and sequencing were done by Novogene (UK) Company Ltd. using a NovaSeq 6000 instrument with S4 flowcell and yielded 7.4 million read pairs (2 × 150 bp). For long-read sequencing, DNA was extracted using a Biosearch Technology Masterpure Yeast DNA Purification Kit (MPY80010). Oxford Nanopore sequencing was done on a MinION MK1C instrument with flowcell FLO-MIN112 (R10.4) and barcoding kit SQK-NBD112-24. NanoFilt (v2.3.0) (5) retained 44,648 reads with quality ≥7 and length ≥1,000 bp (reads N50 = 17,687 bp), which were then assembled using Canu (v2.0.0) (6), followed by five rounds of error correction using NextPolish (v.1.4.1) with the Illumina reads (7).

The UCD805 assembly consisted of 14 nuclear scaffolds (total 12.15 Mb; N50 = 888,568 bp) and the mitochondrial genome (31,651 bp; accession number JAVSEF010000001). Three scaffolds terminate with multiple tandem repeats of the sequence (GTGTAGGATGGT)_n_ at both ends, so they are inferred to be complete chromosomes with telomeres (8). This telomeric repeat was found at 16 scaffold ends, indicating that this yeast strain has 8 chromosomes. BLASTN searches detected rDNA arrays at the ends of three scaffolds (JAVSEF010000003, JAVSEF010000012, and JAVSEF010000013).

The UCD805 genome assembly has average nucleotide identity (9) of 99.75% to the *S. capriottii* type strain NRRL Y-7423 (accession no. JAKTYZ010000000) (10). Using BUSCO version 5.1.2, genome completeness was estimated as 96.83% compared to the Ascomycota lineage data set. G + C content is 34.84%.

## Data Availability

This whole-genome shotgun project has been deposited at DDBJ/ ENA/GenBank accession no. JAVSEF000000000 (BioProject no. PRJNA1018912). The version described in this paper is version 1. The raw reads were deposited at SRA (accession no. SRR26109203 and SRR26109698). The ITS sequence is at OR578565 and the D1/D2 region sequence at OR578569.
